# Relationships and Sex Education Outcomes for Students With Intellectual Disability: Protocol for the Development of a Core Outcome Set

**DOI:** 10.2196/39921

**Published:** 2022-11-07

**Authors:** Laura Paulauskaite, Vasiliki Totsika, Carol Rivas

**Affiliations:** 1 Social Research Institute University College London London United Kingdom; 2 Division of Psychiatry University College London London United Kingdom; 3 Centre for Educational Development, Appraisal, and Research University of Warwick Coventry United Kingdom; 4 Department of Psychiatry Monash University Melbourne Australia

**Keywords:** core outcome set, relationships and sex education, intellectual disability, students

## Abstract

**Background:**

People with intellectual disability are twice as likely to experience sexual abuse, unintended pregnancies, and sexually transmitted diseases as people in the general population. Despite this, very little is known about how to deliver relationships and sex education effectively to this vulnerable population, how to measure the impact of its delivery in schools, and what stakeholders perceive as important outcomes of this education.

**Objective:**

To address these urgent issues, this study aims to develop a stakeholder consensus–based core outcome set of relationships and sex education for use in research and educational settings with students with intellectual disability.

**Methods:**

The study will use a 2-stage mixed methods design. The first stage will involve a systematic review of relationships and sex education outcomes reported in the literature, followed by qualitative exploration with caregivers, teachers and school staff, policy makers, and researchers to investigate their perspectives of meaningful outcomes of this education. Students with intellectual disability will be enabled to take part to express their views on outcomes of importance to them. The second stage will use findings from stage 1 in a 2-round web-based Delphi study with caregivers, teachers and school staff, policy makers, and researchers to develop consensus on proposed outcomes for the evaluation of relationships and sex education with this population.

**Results:**

As of September 2022, we have completed a systematic review and recruited 56 stakeholders (n=53, 95%, adults and n=3, 5%, students with intellectual disability) for the first stage of the study. We are still recruiting students with intellectual disability. Data analysis has not started yet. Recruitment for the second stage will commence in November 2022. We expect to complete the study by October 2023 and publish the results by the end of 2024.

**Conclusions:**

The development of a core outcome set of relationships and sex education will provide a significant first step to assist the implementation, delivery, evaluation, and sustainability of relationships and sex education for students with intellectual disability. Key audiences will be teachers, researchers, policy makers, and decision makers.

**Trial Registration:**

Core Outcome Measures in Effectiveness Trials 1787; https://www.comet-initiative.org/Studies/Details/1787

**International Registered Report Identifier (IRRID):**

PRR1-10.2196/39921

## Introduction

### Background

Approximately 2% of the children in the world have intellectual disability (ID), which is defined as a lifelong neurodevelopmental condition characterized by limitations in cognitive and adaptive skills [1,2]. Children and young people with ID are among the most disadvantaged and vulnerable people in our society [3,4]. They have between 4 and 6 times higher risk of being sexually abused than children without ID [3-5]. Young people with ID are also 2 times more likely to practice unsafe sex and experience sexually transmitted diseases and unintended pregnancies than their peers from the general population matched by age, sex, and exposure to other sociodemographic variables [6]. Although young people with ID are more likely to be experience bullying, some young people with ID can also be bullying perpetrators, possibly because of lack of social skills, difficulties with emotion regulation, or inability to recognize bullying behaviors and other people’s verbal and nonverbal communication cues [7,8]. One potential route to reduce these higher risks is through effective relationships and sex education (RSE) delivered in schools. Despite its importance, little is known about how to deliver RSE effectively to this population; what students with ID should achieve in, and from, RSE lessons; and what students with ID, their caregivers, and teachers perceive as important outcomes of this education.

Evidence from systematic reviews carried out on RSE content, delivery, and effectiveness for people with ID of any age indicates that existing RSE programs do not have clear outcome goals, and the outcomes measured lack consistency [9-12]. Heterogeneity in RSE outcome reporting makes it challenging to compare the effectiveness of RSE across studies, and this affects the development of appropriate evidence-based RSE for this vulnerable population. Furthermore, these reviews highlight that people with ID are not involved in the development of RSE programs, and thus the content delivered and outcomes measured do not reflect their views [9-12]. This might possibly lead to ineffective or even harmful RSE programs delivered to students with ID—for example, if there are unexpected adverse outcomes that could have been anticipated by working with them—and research waste.

The development of a core outcome set (COS) could help to address these limitations in the current evidence base. The COS involves identifying *what* to measure and includes a consensus of stakeholders’ opinions on what could constitute meaningful outcomes [13]. The COS provides a minimum standard of outcomes that all randomized controlled trials, evaluation studies, and practice-based audits should measure and report within a specific health or social care area [14]. This standardization of outcomes improves research utility by involving stakeholders’ perspectives as well as reducing inconsistency, reporting bias (when only preferred outcomes are reported instead of all outcomes assessed), and research waste [14]. The Core Outcome Measures in Effectiveness Trials (COMET) Initiative has developed a standardized methodology that has been successfully used to develop a COS across a wide range of health and social care areas [14]. However, there is no published COS of RSE for students with ID. The development of such a COS will not only help to demonstrate different perspectives and develop much needed consensus in this sensitive area but also provide, for the first time, a standardized set of outcomes to be used in research and educational practice to assess RSE delivery and help to design and develop the curriculum or evaluation studies.

### Aim and Objectives

The aim of this project is to develop a stakeholder consensus–based COS of RSE for students with ID. The specific objectives of the study are as follows:

- Develop a comprehensive list of potential outcomes through (1) existing evidence on RSE outcomes for students with ID reported in the literature and (2) data collected from key stakeholders, including students with ID, caregivers, teachers and school staff, policy makers, and expert researchers.
- Finalize a COS using a structured consensus-based approach.

## Methods

### Ethics Approval

Ethics approval for the study was received from the research ethics committee of the Institute of Education, University College London (REC 1565).

### Design

**Figure 1:**
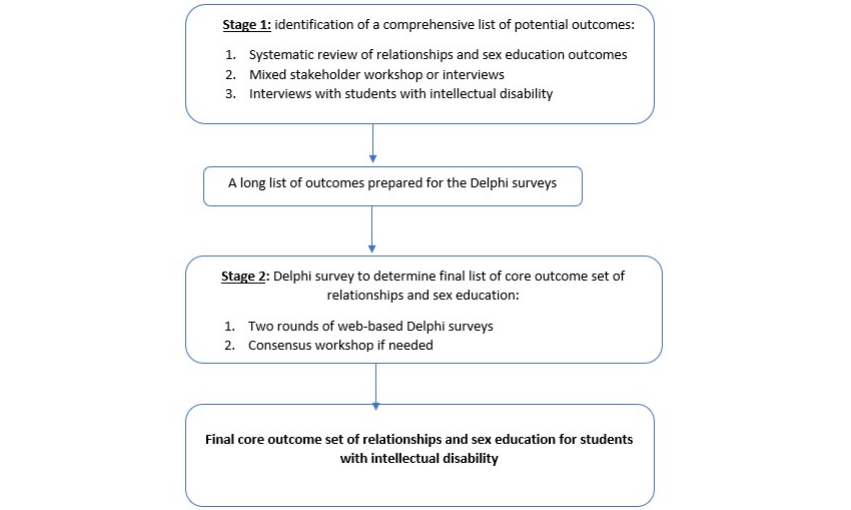
Study design.

The COMET handbook for COS development [14] will guide the methodology of this study. The study protocol has been written following the Core Outcome Set–Standardized Protocol Items guidelines for reporting protocols of COS development [15]. The study will use a 2-stage mixed methods design that involves a systematic review, stakeholders’ workshops and interviews, a Delphi web-based survey, and, if needed, a subsequent consensus workshop (Figure 1).

### Scope of the COS

#### Population

The COS of RSE for students with ID will be developed for use in English educational settings and research evaluations primarily in Western countries, with a potential extension to non-Western countries after adaptations that reflect their cultural, socioeconomic, and policy characteristics.

Our target population is students aged 5 to 25 years with any level of ID (mild, moderate, severe, or profound) and verbal ability (verbal, minimally verbal, or nonverbal). Children with ID are a heterogeneous group with a wide range of abilities and additional needs. ID is a neurodevelopmental condition characterized by significant limitations in intellectual functioning and adaptive skills present from the developmental period (0-18 years) [2]. Intellectual functioning involves activities such as learning and abstract thinking. Adaptive skills refer to conceptual, social, and practical skills used in everyday activities such as socializing or feeding oneself. ID is diagnosed by a score of 2 SDs below the general population mean on standardized tests of cognitive skills—for example, IQ <70—*and* adaptive skills score <70 [2]. ID can be classified into 4 levels depending on severity: mild, moderate, severe, and profound. Children with mild ID may have subtle developmental delays (eg, they may have school learning problems and delays in communication abilities), whereas children with severe or profound ID will have severe developmental delays (eg, they may be nonverbal and require significant support with basic needs such as using a toilet or feeding) [2]. ID can be caused by genetic and chromosomal abnormalities (eg, Down syndrome and Fragile X syndrome, which can be associated with having more severe ID) or problems during pregnancy and birth (eg, infection or maternal substance abuse) [2]. Many children with ID also have co-occurring conditions such as autism and sensory processing difficulties as well as mental and physical health problems [16,17]. Therefore, students with any level of ID and co-occurring conditions will be eligible to take part in the study, ensuring that this COS is applicable across the ID spectrum.

In England, the term *intellectual disability* is synonymous with *learning disability or difficulty*, which is a term that some study participants might recognize more easily. Our target population will include students who have a formal ID diagnosis as well as students whose ID is administratively defined (eg, those who receive special education or other services because they have ID). The age range was selected to include all school students with ID who are receiving RSE. In England, RSE was made compulsory in all schools in 2020 [18]. RSE begins in primary schools (when students are aged 5-11 years) and continues through to secondary schools (when students are aged 11-16 years) [18]. However, the upper age limit of 25 years was selected because some students with ID in England remain in school education until the age of 25 years [19].

#### Intervention

Our target intervention is RSE that is a curriculum delivered directly to students in schools or other educational settings. Different terms across countries globally are used to refer to RSE (eg, *sex education* or *reproductive health education* or *life skills education*) [20]. RSE terminology, delivery, content, and policy also differ across the United Kingdom. We use *RSE* because it is the preferred term in England, the setting of the study. The statutory guidance on RSE delivery in England indicates that students in primary schools must receive education on topics regarding positive and unhealthy relationships, and in secondary schools, they must receive information on sexual relationships, sexuality, and safeguarding (eg, exploitation and female genital mutilation) [19]. The RSE content delivered to students with ID can be adapted to their developmental levels [19].

RSE can be delivered as a stand-alone subject or integrated into other subjects [21]; for example, in England, RSE is usually delivered within the wider curriculum of personal, social, health, and economic education [21]. RSE can be delivered by teachers, school-based nurses, or external specialists [22]. It is often delivered to a whole classroom, but for students with ID, RSE may be provided in small groups or on a one-to-one basis in social care settings (eg, at day centers or group homes) and at students’ homes (eg, after-school activities delivered by caregivers at home) in addition to being delivered at schools [23].

#### Stakeholders and Setting

We will involve 5 groups of stakeholders in the development of this COS: (1) students with ID; (2) caregivers of students with ID (eg, parents or other unpaid individuals who are responsible for providing care to the students); (3) teachers and school staff who are involved in the delivery of RSE to this population; (4) policy makers such as people from government agencies, parliamentary committees, third sector organizations, and charities that support families of children with ID and specialize in special education policy in England, as well as those who were also involved in formulating the RSE policy in England; and (5) researchers who have authored key papers on RSE for people with ID or specialize in research on the sexuality and relationships of people with ID or education for people with ID. Students with ID and their caregivers as well as teachers and school staff will be approached via established mainstream and special school networks that serve students with ID in England. We will aim to recruit stakeholders from mainstream and special schools because both settings serve students with ID in England, which might allow us to capture a variety of opinions on important outcomes. Expert researchers (from England and internationally) will be identified from the systematic review (refer to the Systematic Review section), internet searches, and snowball recruitment. Policy makers will be identified through special education charities and policy networks in England, internet searches, and snowball sampling.

We will recruit 10 to 15 participants in each of the four stakeholder groups (caregivers, teachers and school staff, researchers, and policy makers) for the data collection from adults using 2 data collection methods (ie, for both the workshop and interviews combined) and 10 to 15 students with ID for the interviews. For the Delphi web-based survey, we will recruit 10 to 15 participants in each of the 4 stakeholder groups (caregivers, teachers and school staff, researchers, and policy makers). There is no *gold standard* number of stakeholders to involve in the COS development and Delphi surveys [13]. However, consensus approaches with 15 participants are considered to be effective [24]. We will pilot study materials to check clarity and ambiguity with stakeholders who are not taking part in the study where possible.

### Stage 1: Identifying a Comprehensive List of Potential Outcomes

In stage 1, potential outcomes will be identified through a systematic review, a mixed stakeholder workshop, interviews with adults, and interviews with students with ID.

#### Systematic Review

The review protocol was registered prospectively with PROSPERO (CRD42021243176), and the findings were published elsewhere [25] before our primary data collection. The objectives of the review were to (1) identify outcomes of RSE for students with ID reported in existing studies, (2) identify the measurement instruments used to measure RSE outcomes, and (3) evaluate the identified instruments’ measurement properties (validity, reliability, and responsiveness) using the Consensus‐Based Standards for the Selection of Health Measurement Instruments criteria [26] that were developed for evaluating the quality of COS measurement instruments.

The search consisted of 2 parts. The first part of the search was carried out in March 2021 to identify all RSE outcomes and their measurement instruments published in any language using 9 electronic databases (eg, MEDLINE, Embase, and PsycINFO) and gray sources (eg, ResearchGate and Google Scholar). The second part of the search was carried out in August 2021 to retrieve studies on the identified instruments’ measurement properties using the same databases and gray sources as in the first part [25]. Outcomes identified through the review were extracted verbatim from the text and grouped into 3 separate lists based on the students’ age. These lists of potential outcomes will be incorporated into the data collection methods (the workshop, interviews, and Delphi survey) with key stakeholders for their consideration.

#### A Mixed Stakeholder Workshop

The process of a priority setting partnership based on the James Lind Alliance principles [27] will be adapted to carry out a mixed stakeholder workshop (carried out either face to face or remotely depending on participants’ preferences and the extant COVID-19–related regulations) with caregivers, teachers and school staff, policy makers, and researchers to gather information on what outcomes they perceive as necessary and important for RSE. The priority setting partnership is a structured consensus-based approach that involves mixed stakeholder groups working together to produce a prioritized list of outcomes [27]. Before stakeholders are invited to attend the workshops, we will obtain informed consent for participation. Demographic information (such as participants’ ethnicity and religious affiliations) will be collected and used when analyzing the results because research indicates that people from different cultural and religious backgrounds hold different attitudes toward the sexuality of people with ID as well as different beliefs on what topics are appropriate to deliver to students with ID in RSE [28-31].

At the workshops, stakeholders will be split into smaller groups (6-8 in each group; there will be a caregivers’ group, teachers’ group, etc). Each group will be asked to discuss a list of possible outcomes of RSE. Prompts (eg, pictures) will be provided to facilitate the discussion among the groups, if needed. Each group will be asked to come up with the top 3 outcomes of RSE, which will be written down on a board or flip chart for everyone to see. When presenting their top 3 outcomes each group will be asked why they have chosen these outcomes and how important they are to the group, and this will be discussed with the wider group. The lists of outcomes extracted from the systematic review will also be available for participants to see. The workshops will be audio recorded, transcribed in full, and analyzed. The recording of the smaller group discussions might provide additional outcomes that were discussed in the smaller groups but were not presented for the whole group discussion, perhaps because the outcome was mistakenly not thought feasible and measurable by the smaller group. This method will allow us to explore differences among the subgroups (eg, what outcomes the caregivers perceive as the most important) while unpacking their different understandings together and also achieving a consensus-based list of outcomes at 1 workshop, thus reducing the demand on participants.

Because of the research topic that may be perceived by some participants as sensitive or uncomfortable for a discussion in a group format, we will be offering to these participants the opportunity to express their views on RSE outcomes in a one-to-one semistructured interview. The interviews will follow the same procedure as described for the workshop, but they will be conducted individually and will be of shorter duration than the workshop.

#### Interviews With Students With ID

##### Overview

The views of students with ID on RSE outcomes (identified through the systematic review, workshop, and interviews with adults) will be explored using individual face-to-face interviews conducted by the main researcher (LP). Interviews were selected because many students with ID have complex needs and differences in their communication profiles. Face-to-face sessions have been chosen because research conducted on remote sessions with children with ID during the COVID-19 pandemic indicates that the children found it very challenging or impossible to engage in web-based sessions [32]. Moreover, teachers at special schools reported that approximately 30% of the families of children with ID had no access to a computer or the internet during the pandemic [32]; thus, a remote approach would exclude many voices.

The interviews will use 1 of 3 promising visual qualitative data collection methods catering to different student communication profiles and abilities: (1) a picture-sorting activity based on the Talking Mats framework, (2) an art-based session, or (3) a diamond ranking activity (for more details, refer to the sections that follow and Multimedia Appendix 1 [33-35]). The choice of method for a specific student will be based on their prior experience of using a similar method and preferences. Three data collection methods will be offered because the literature indicates that a *one-size-fits-all* method for exploring the views of this heterogeneous population is not effective [36,37]. Preparatory sessions with the participating students’ caregivers or teachers before the interviews will be conducted by the main researcher (LP) to discuss prior familiarity with the proposed measures, proposed adaptations to the interview to accommodate students’ needs, and likely cultural restrictions regarding the topics selected. The interviews will be piloted with a small group of students who will not be taking part in the study. All interviews with study participants will be audio recorded because students with verbal abilities might provide their views of RSE outcomes or might suggest additional outcomes. Students’ teachers or caregivers will be present (if the child does not object) at the interview with the main researcher (LP). Pictures will be taken of completed activities (eg, sorted mats of RSE outcomes). Details of the 3 visual data collection methods have been provided in the following sections.

##### A Picture-Sorting Activity Using Talking Mats

Talking Mats have been shown to be effective in enabling people with different levels of ID and verbal communication abilities to express their views [33,38,39]. Talking Mats is a visual, structured, symbol-based communication framework that allows a person with verbal difficulties to express their views by using symbols [33]. In this activity, students will be introduced to the RSE topic (eg, “We are going to talk about what you learn at school. Tell me which topic you like.”) and asked to place pictorial RSE outcomes under categories of the visual scale (eg, *OK* or *Do not know*) adapted for each student. The ranking of outcomes will start with practice rounds of sorting neutral topics, making sure that the students understand the task, and building up toward RSE outcomes. At the end of the sorted RSE mat, the interviewer will discuss the placements of pictures to double-check that the sorted pictures correspond to the students’ views and were not sorted randomly.

##### Art-Based Session

Art-based methods (eg, drawings and posters) have been used in educational and therapeutic settings to explore the views of people with ID who struggle to express themselves verbally [34,40]. This method was selected because students do not need to have good expressive language or understand a visual scale (eg, *OK* or *Do not know*) to take part. In this activity, students will be presented with art and craft material of different textures (eg, pictures of RSE outcomes coproduced with students with ID, sticky notes, and water paint) as well as a large piece of paper and asked to make a *what I want to learn about growing up* poster (adapted to each student’s comprehension and RSE level). An example of how to perform this activity will be presented, and students will be guided through the process whenever needed. Students with verbal abilities will be asked simple questions about what they selected and why. Carers of students with limited or no verbal abilities will be asked to comment on what the student selected and what they think are the reasons for the student’s choice.

##### A Diamond Ranking Activity

A diamond ranking activity involves ranking items from most important to least important in a diamond shape [35] and has been used successfully previously with young people with ID and complex communication profiles [41-43]. This activity might be particularly suitable for students with ID who do not like direct questioning because the emphasis in this activity is on sorting items in the diamond shape, and questions can be asked while students complete the task [41]. In this activity, students will be presented with pictures of RSE outcomes that will have short verbal descriptions at the bottom of each picture. Students will be asked to sort the pictures of RSE outcomes by placing pictures on a piece of paper in a diamond shape with examples provided as needed. They will be told to place outcomes of RSE that they like at the top, the outcomes that they are unsure about in the middle row, and the outcomes that they do not like at the bottom. Students will be asked questions about their rating choices and their views on outcomes.

##### Coproduction of Pictorial RSE Outcomes

In this study the pictorial RSE outcomes (identified in the systematic review, workshop, and interviews with adults), coproduced with students with mild ID (n=5) who are not taking part in the interviews, will be used with participating students with ID in the interviews (eg, diamond ranking activity). The main researcher (LP) will present different pictures to the students (selected from pictorial databases designed for people with ID, such as Photosymbols [44], and pictures taken by the main researcher of RSE teaching materials that schools use). The main researcher will ask the students to select the most accurate pictures to represent RSE outcomes using the Talking Mats framework. The coproduction of material with people with ID using Talking Mats has been undertaken successfully previously [45]. This coproduction of pictorial RSE outcomes has the aim of ensuring that the symbols chosen to represent RSE topics are appropriate and are not confusing for students with ID; thus, they do not lead to misinterpretations.

### Data Analysis

Qualitative data collected from the mixed stakeholder workshop, interviews with adults, and interviews with students with ID will be analyzed by the main researcher (LP) using the reflective thematic analysis approach described by Braun and Clarke [46]. Precisely 20% of the data will be second-coded by another researcher for dependability and confirmability. Interrater reliability will be measured using the Cohen κ coefficient and based on the parameters proposed by Landis and Koch [47]. Quantitative data—for example, diamond ranking activity—will be analyzed using descriptive statistics such as medians and percentages.

### Outcome Generation

We will follow the COMET guidelines [14] for organizing the outcomes identified in stage 1 of the project. All outcomes identified from the systematic review, workshop, interviews with adults, and interviews with students with ID will be extracted verbatim, compiled by the main researcher (LP) into three long lists based on students’ age (eg, RSE outcomes for primary education reported for, or by, students with ID aged 5-11 years; RSE outcomes for secondary education reported for, or by, students with ID aged 11-16 years; and RSE outcomes for further education reported for, or by, students with ID aged 16-25 years). In these 3 lists, outcomes that are overlapping will be deduplicated by the main researcher (LP), and outcomes considered semantically related by the main researcher (LP) will be presented to the senior research team (CR and VT) and, after a discussion, merged into outcome domains. The senior research team will review outcomes that were categorized into 3 lists. These 3 long lists of possible RSE outcomes will be presented in stage 2 of the project, the Delphi survey to reach consensus on the final COS. The list of outcomes identified from the systematic review, workshop, and interviews with adults and students will be incorporated into the web-based survey.

### Stage 2: Delphi Survey to Determine Final List of COS of RSE

#### Web-Based Delphi Survey

The web-based Delphi process will be used to reach consensus on the COS. The process involves completion of web-based questionnaires answered anonymously by a panel of stakeholders (caregivers, teachers and school staff, policy makers, and expert researchers) [48]. The web-based survey will be first piloted with a small number of caregivers, teachers, or other professionals who are not taking part in the project to assess clarity and readability before being sent to the Delphi survey participants. Before they take part in the survey, participants will receive information about the study, explaining how to rate the outcomes and the importance of completing surveys in both rounds. Participants will also be offered support from the research team if they have difficulties completing the survey. The survey will be administered using the Qualtrics web-based survey tool [49], and participants will receive individual invitations to the survey via email. When participants click on the survey link, they will be asked to provide informed consent to take part in the survey and answer demographic questions embedded in the survey.

In round 1, stakeholders will be presented with the outcomes (identified through the systematic review, workshop, and interviews with adults and students) and asked to score anonymously the importance of including a particular outcome in a COS on a 9-point Likert scale using a scoring framework recommended by the Grading of Recommendations Assessment, Development, and Evaluation [50]. On the basis of this framework, outcomes scored 1 to 3 will indicate *not important* outcomes, 4 to 6 will indicate *important but not critical* outcomes, and 7 to 9 will indicate *critical* outcomes. Participants will also have an option to choose *unable to score* for items and provide comments and feedback in free-text boxes for each rating question.

The plan is for all outcomes from round 1 to be retained in round 2 to allow stakeholders to see each group’s ratings and then make a final decision regarding the importance of including this outcome in the COS. However, we will consider dropping outcomes after round 1 to reduce participant burden and attrition rates in round 2 if the piloting of the survey indicates that the initial list of potential outcomes is considered by stakeholders to be too long. In this case, an outcome from round 1 will be retained in round 2 if ≥50% of the participants rate it with a score of 7 to 9 and <15% of the participants rate it with a score of 1 to 3. An outcome will be dropped if ≥50% of the participants rate it as 1 to 3 points and <15% of the participants rate it as 7 to 9 points. New outcomes suggested by participants in the comments sections will be included in round 2.

Participants who complete at least 75% of the survey in round 1 will be invited to participate in round 2. In round 2, stakeholders will be able to see a summary of scores of each stakeholder group presented separately as a median score. Each person will be asked to think about the group results and decide whether they want to change their responses. Outcomes will be analyzed using descriptive statistics in Excel (Microsoft Corporation). Consensus to retain an outcome in the final COS list will be determined to have occurred if ≥70% of the respondents score it as 7 to 9 points and <15% of the respondents score it as 1 to 3 points. Outcomes that are scored by ≥70% of the respondents as 1 to 3 points and by <15% of the respondents as 7 to 9 points will not be included in the final COS list. Attrition bias will be assessed by comparing the average scores of each outcome rating of participants in each stakeholder group who complete only round 1 with the average scores of the participants who complete both rounds to see whether this affects the final COS list.

In both rounds, participants will have 2 weeks to complete the survey, and participants who have not completed the survey and have not declined to participate will receive 2 email reminders during this period.

#### Optional Final Workshop

Stakeholders from the Delphi survey will be invited to a final workshop (which will either be held face to face or on the web depending on participants’ preferences and the extant COVID-19–related regulations) if all outcomes identified as critical by students with ID (as they will not take part in the Delphi survey) did not end up being included in the final COS or if there are a large number of outcomes that participants are unable to reach consensus on. In this final workshop, stakeholders will be presented with these omitted outcomes as well as outcomes on which there was no consensus and asked to discuss and reconsider them. If, even after the final workshop, all outcomes identified by students with ID as critical do not end up being included in the final COS, we will report these outcomes as well as an overview of the workshop discussion and recommend that users of a COS of RSE add at least one outcome from the list identified by students with ID.

### Dissemination Plan

The findings will be published in academic journals (eg, *American Journal on Intellectual and Developmental Disabilities* and *Journal of Intellectual Disability Research*), registered at the open-access COMET database, presented at special schools using links that the team has with special schools and research networks, and presented at conferences. An easy read summary of findings will be disseminated to the different stakeholder groups involved. Through these dissemination events, we will encourage schools that serve students with ID to adopt the COS to evaluate their current delivery or to work with researchers to achieve this. We will publish a policy briefing on the final COS so that the findings reach a wider policy maker audience. We will contact relevant journals and funding bodies so that they may include the COS in their guidelines for researchers and authors.

## Results

As of September 2022, we have completed a systematic review and recruited 56 stakeholders (n=53, 95%, adults and n=3, 5%, students with ID). We are still recruiting students with ID for the interviews. Data analysis has not started yet. Recruitment for the Delphi survey will commence in November 2022, and recruitment for the additional workshop (if needed) will be carried out in January 2023. We expect to complete the study by October 2023 and publish the results by the end of 2024.

## Discussion

### Anticipated Findings and Potential Impact

This protocol describes a study that aims to develop the first COS of RSE with, and for, students with ID aged 5 to 25 years. The findings from the study have the potential to have immediate application in English educational practice and policy because it is little known what students with ID should achieve in compulsory RSE lessons and how to obtain caregivers’ support for RSE’s aims and objectives. It will provide information of importance to researchers by proposing a standardized set of outcomes that should be measured in all RSE evaluation studies; this will enable the building of an evidence base for RSE programs for students with ID in Western countries.

This study will also provide theoretical and conceptual developments in the engagement and perspectives of a range of stakeholders, including students with ID, their caregivers, teachers, researchers, and other experts. A flexible approach will be used to engage students with ID of varying communication needs and ID severity level to ensure that they are included in the study and that their opinions are incorporated.

### Comparison With Prior Work

Previous systematic reviews on RSE programs delivered to people with ID indicate inconsistent outcome measurement in evaluation studies and a lack of involvement of people with ID in the development of such programs [9-12]. Therefore, our work will address these gaps in the evidence. The proposed COS will include the views of students with ID and other key stakeholders and will be based on consensus across groups. Crucially, this will be the first COS of RSE for students with ID and, to our knowledge, the first COS on any topic developed for, and with, people with ID. This COS is also one of a few COS lists developed for children and young people that involves them in the development as participants and uses data collection methods adapted to the population [51,52].

### Strengths and Limitations

The key strength of the study is the aim to involve different stakeholders in the process and, most importantly, students with ID, which has never been done before in COS development. We will also use multiple data collection methods (eg, systematic review, workshop, interviews, and Delphi survey) to gather different perspectives on this topic. Three data collection methods will be used with students with ID to support research participation by students with different abilities.

Although the empirical work supporting this COS will be limited to a specific geographical and cultural context (England) and to the specific group of students with ID to be recruited (aged 5-25 years), the systematic review supporting this COS considered the international literature [25]. Thus, although the COS findings will be mostly generalizable to this area and this population, we are incorporating evidence from all available international studies in the first step toward building this COS.

This COS of RSE will be developed with, and for, students with ID, who make up a heterogeneous population with varied abilities, needs, and copresenting problems. Our inclusion criteria for the students and other key stakeholders to take part in the study are wide. We are not excluding students based on their level of ability or other co-occurring conditions (eg, autism) because an inclusive group of participants is more representative of the group of students with ID currently being educated in English special and mainstream schools. We are also including other stakeholders (eg, caregivers, teachers, and researchers) who support students with a range of abilities because each of them will bring a unique and valuable perspective. However, the empirical work supporting this COS will be based on convenience sampling and will not be generalizable to all students with ID in all areas. Therefore, the outcomes included in this COS will need to be adapted when applied in practice in different contexts to take into account a student’s location, culture, developmental level, and current RSE knowledge. Adaptation of RSE measurement will be important in future applications of this COS to better reflect the profile and needs of the population being measured. However, it is also extremely important that the selection of outcomes is not based on a selector’s individual beliefs and societal attitudes. The outcomes excluded from measurement for a particular student at a particular point in time will need to have comprehensive justification, making sure that a student’s level of ID (which determines intellectual functioning and adaptive skills, not their sexual and relationship needs) is not the sole reason for the omission of outcomes.

In the course of the proposed study, it is likely that our inclusive recruitment plans might face difficulties; for example, we might struggle to engage directly with a specific subgroup of students such as those with severe ID and stakeholders from diverse cultural and religious backgrounds. Past research indicates that recruitment to the studies on this topic can be challenging because of the topic being perceived as sensitive or taboo [53-55]. Studies also indicate that in some cultures it is shameful to talk about the sexuality of people with disabilities [31,55-57] and that some stakeholders hold beliefs that people with severe ID do not need to receive RSE [58]. These attitudes may affect recruitment to the study. Our data collection methods might also fail to enable all students with ID to express their views. The COVID-19 pandemic might also affect the availability of school staff and caregivers to take part in the study. Therefore, we might fail to capture perspectives of specific subgroups of stakeholders in this COS, and future work might be needed to address these gaps if this occurs.

### Conclusions

There is a high need for a COS of RSE to guide the development and provision of RSE curricula in special and mainstream education for students with ID. This COS of RSE aims to engage different stakeholders in the process of its development and achieve a consensus on the core RSE outcomes that are important for this population. The findings have the potential to improve current RSE practice in English educational settings, harmonize RSE outcome measurement in research, and support the development of effective RSE programs for students with ID in Western countries.

## Appendix

Multimedia Appendix 1 Summary of methods to be used with students with intellectual disability.

## Abbreviations

COMET: Core Outcome Measures in Effectiveness Trials
COS: core outcome set
ID: intellectual disability
RSE: relationships and sex education

## Data Availability

Data sharing will not be available because this study considers sensitive information. It was not a requirement by the funder to deposit data in a publicly available repository for studies undertaken by PhD students, and participants’ consents for data sharing were not obtained in this study.
