# Supplementary material for: Relationships and Sex Education Outcomes for Students With Intellectual Disability: Protocol for the Development of a Core Outcome Set
Source: JMIR Res Protoc. 2022 Nov 7;11(11):e39921. doi: 10.2196/39921 (PMC9679930; doi:10.2196/39921)
Supplement: Multimedia Appendix 1 [file resprot_v11i11e39921_app1.docx]

## Supplementary material

Table 1: Summary of methods to be used with students with ID

| **Method** | **What involves** | **Suitability** | **Advantages** | **Disadvantages** |
| --- | --- | --- | --- | --- |
| 1. Picture sorting activity using the Talking Mats [33] | Placing pictorial outcomes of RSE under “ok” and “I do not know” categories or other visual scale that a student is familiar with e.g. “need” and “do not need”.  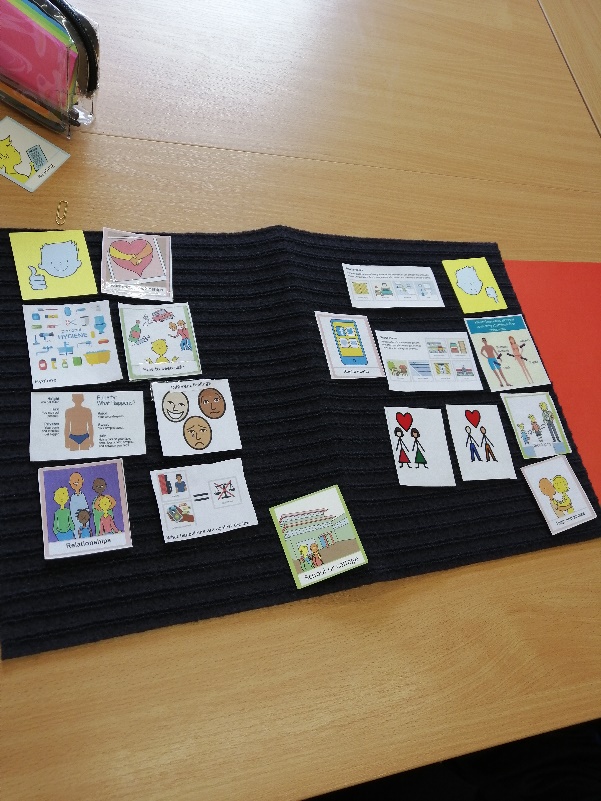 | -used picture exchange systems before or likes using pictures when communicating  - able to recognise pictures  -able to make a choice  - good eye hand coordination for moving the pictures and fine motor abilities for picking up the pictures from the mat or pointing to the pictures  - has good receptive language (e.g. understands simple questions or 2 word phrases) but no need of having expressive language (ability to talk) | -flexible (students can go on their own pace and take breaks and go back and change their minds)  - does not need to be completed sitting at the table and could be completed hanging the mat on the wall so the student could move in the room (this might be suitable for students with ID and ADHD)  -provides views of students who might not be able to verbally express themselves  -if used before, might be familiar or similar activity  -hands-on approach | - Students who never used Talking Mats might find difficult to take part  -Students who cannot make a choice might find difficult to take part |
| 2. Arts-based session [34] | Using what a student likes e.g. pictures, drawings, sticky notes or “Play Dough” to produce a “what I want to learn about growing up” poster or collage or just individual images. Simple questions during this session will be asked to guide the expression of views on RSE topics.  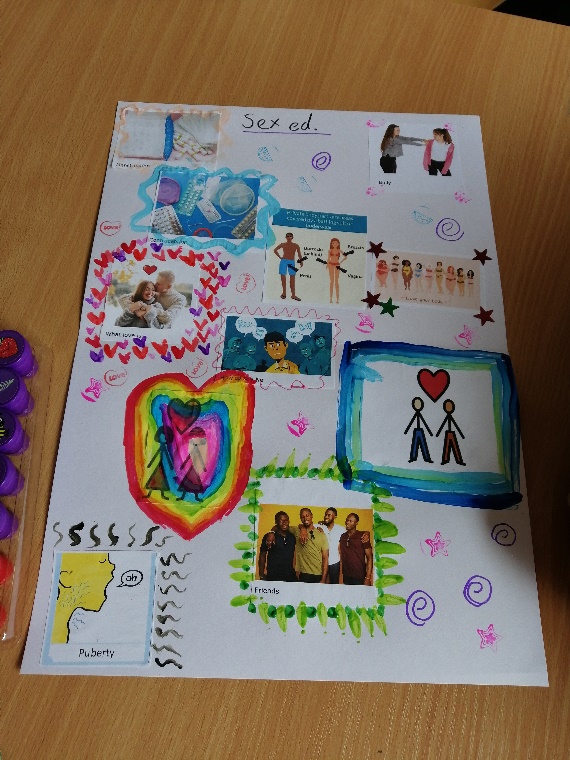 | - no need to have expressive language but a minimal receptive language (2-word phrases)  - no need to be able to understand a visual scale e.g. “like” and “do not like”  - ability to recognise pictures if pictures are selected for the task  - good fine motor abilities for picking up the pictures and placing on a poster if pictures selected for this activity and for drawings if drawings are used  - could be completed sitting on a table or hanging the poster on a wall | - fun, creative, sensory, hands-on activity  -flexible  -provides views of students who might not be able to verbally express themselves | - if a student does not have verbal communication skills it might be difficult to understand what has been produced or whether this is about RSE thus the reliability of data might be questionable.  - might need help from a student’s carers to help facilitate the communication for students that are non-verbal |
| **Method** | **What involves** | **Suitability** | **Advantages** | **Disadvantages** |
| 3. Diamond ranking activity [35] | Students will be asked to sort pictures that represent RSE outcomes into a diamond shape to express their views. For example, they will be told to place outcomes of RSE that they like on the top (row 1), the outcomes that they are unsure about in the middle row (row 2), the outcomes that they “do not like” to place on the bottom (row 3) and will be asked about their ranking choices (e.g. “Why this picture was placed on top of the diamond?”)  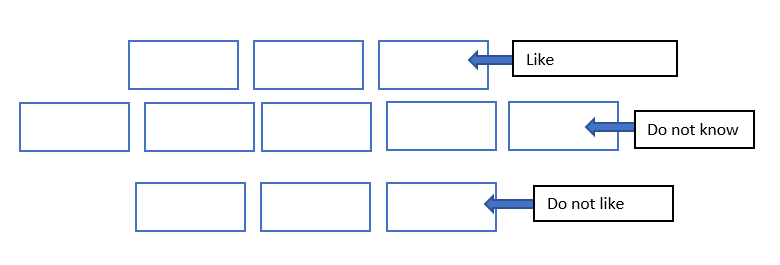 | - good expressive and receptive language  -ability to understand simple questions  -good reasoning abilities  - for students who can engage in one-to-one discussions  -for students who do not like direct questioning (e.g. standard interviews) but can answer questions while doing an activity | - instead of asking direct questions in an interview format, this is a hands-on approach  -might be more suitable for students with ID and autism who do not like direct questioning  - would provide students with ID views of RSE and ranking of the outcomes  -for students who do not like using Talking Mats and prefer answering questions | - students with no expressive language and reasoning abilities will not be able to take part but there are other methods suggested |
